# Effect of early dose reduction of osimertinib on efficacy in the first-line treatment for *EGFR*-mutated non-small cell lung cancer

**DOI:** 10.1007/s10637-024-01432-4

**Published:** 2024-03-27

**Authors:** Tomoki Hori, Kazuhiro Yamamoto, Takefumi Ito, Shigeki Ikushima, Tomohiro Omura, Ikuko Yano

**Affiliations:** 1 Department of Pharmacy, Nara Prefecture General Medical Center, 2-897-5 Shichijo-nishimachi, Nara, 630-8581 Japan; 2 Department of Pharmacy, Kobe University Hospital, 7-5-2 Kusunoki-cho, Chuo-ku, Kobe, 650-0017 Japan; 3 Department of Respiratory Medicine, Nara Prefecture General Medical Center, 2-897-5 Shichijo-nishimachi, Nara, 630-8581 Japan

**Keywords:** Osimertinib, Dose reduction, Epidermal growth factor receptor, Progression-free survival, Non-small cell lung cancer

## Abstract

Osimertinib is used as the first-line therapy for patients with *epidermal growth factor receptor* (*EGFR*)-mutated non-small cell lung cancer (NSCLC). However, early dose reduction is often required due to adverse events (AEs). This study aimed to evaluate the effect of early dose reduction of osimertinib on efficacy and safety. This was a retrospective study including patients with *EGFR*-mutated NSCLC who were started on osimertinib as the first-line therapy between August 2018 and December 2021. Patients whose doses were reduced to less than 80 mg/day within 6 months of osimertinib initiation or started at 40 mg/day were defined as the dose reduction group. The primary endpoint was progression-free survival (PFS). Factors affecting PFS were explored using the Cox proportional hazards model. A total of 85 patients were included in this study. No significant differences in patient characteristics were observed between the dose reduction (*n* = 25) and standard dose groups (*n* = 60). The median PFS in the dose reduction group was significantly prolonged compared with that in the standard dose group (26.0 months vs. 12.0 months, *p* = 0.03). Multivariable analysis of 84 patients, excluding a patient with unknown brain metastasis, revealed that *EGFR* exon 21 L858R mutation, malignant pleural effusion or pleural metastasis, liver metastasis, and dose reduction within 6 months were independent factors affecting PFS. Early dose reduction of osimertinib is an effective therapeutic strategy for prolonging PFS in patients with *EGFR*-mutated NSCLC.

**Supplementary Information:**

The online version contains supplementary material available at 10.1007/s10637-024-01432-4.

## Introduction

Lung cancer is one of the most aggressive tumors and is a leading cause of death from cancer worldwide. Non-small cell lung cancer (NSCLC) is the most common type of lung cancer, accounting for approximately 85% of lung cancer cases [1]. *Epidermal growth factor receptor* (*EGFR*) gene mutation is one of the common genetic mutations in NSCLC [2]. EGFR-tyrosine kinase inhibitors (TKIs) provide better efficacy and longer survival than conventional cytotoxic chemotherapy in patients with *EGFR*-mutated NSCLC [3]. A phase III randomized, double-blind, multicenter, international study, the FLAURA study, showed that third-generation TKI osimertinib significantly improved progression-free survival (PFS) compared with first-generation TKI gefitinib/erlotinib [4]. Based on the FLAURA study, osimertinib is commonly used as the first-line EGFR-TKI therapy in patients with a performance status of 0 or 1. However, osimertinib has a predictable adverse event (AE) profile because it inhibits EGFR. Interstitial lung disease (ILD), gastrointestinal disorders such as diarrhea, and skin disorders such as rash/acne, dry skin, and paronychia are the major treatment-related AEs [4, 5]. Among the Japanese subset of the FLAURA study, AEs were noted in 100% (any grade) and 47.7% (grade 3 or higher) of the patients who received osimertinib therapy [5]. Even with the occurrence of AEs, except for moderate and severe ILD, patients can resume treatment with a reduced dose (40 mg/day) only if AE improvement is observed after the withholding of osimertinib [6]. In the FLAURA study, dose reduction due to AEs occurred in 13.8% of the Japanese patients [5] and 5% of the overall patients [7]. This indicates that the osimertinib dose may not be well tolerated in some ethnic groups.

Multiple large-scale studies, such as LUX-Lung7 [8] and RealGiDo [9], have reported an association between efficacy and dose reduction in the first 6 months due to AEs for the second-generation TKI afatinib. However, the FLAURA [4] and OSI-FACT studies [10], which used osimertinib as the first-line treatment, did not adequately examine the association between early dose reduction and efficacy in all age populations, and the feasibility of dose reduction has become a pressing issue for clinicians when their patients experience osimertinib AEs.

Therefore, this study aimed to evaluate the effect of early dose reduction of osimertinib as the first-line therapy on efficacy and safety in patients with *EGFR*-mutated NSCLC.

## Materials and methods

### Patients

This was a retrospective study. Patients with pathologically confirmed advanced or recurrent *EGFR*-mutated NSCLC who were started on osimertinib as the first-line therapy between August 2018 and December 2021 at the Nara Prefecture General Medical Center, Nara, Japan, were enrolled in this study. The exclusion criteria included patients concurrently using other anticancer agents and having an unknown number of TKI therapeutic days. Pleural metastasis was defined as malignant pleural effusion, infiltration, and dissemination. The patients received osimertinib (80 mg/day, 40 mg/day, or less) until the detection of progressive disease or intolerable toxicity. Patients who were started on osimertinib at 80 mg/day and had no dose reduction within 6 months were included in the standard dose group, and those whose doses were reduced to less than 80 mg/day within 6 months from osimertinib initiation or started at 40 mg/day were included in the dose reduction group. This study was conducted in accordance with the provisions of the Declaration of Helsinki and approved by the Institutional Review Board of Nara Prefecture General Medical Center (Approval No. 710). Informed consent was obtained by allowing each patient to opt out of the enrollment in this study at any time by reviewing the study summary published on the website.

### Endpoints

The primary endpoint was PFS. The secondary endpoints were prognostic factors affecting PFS, time to treatment failure (TTF), overall survival (OS), and severity of AEs before and after dose reduction in the dose reduction group. PFS was defined as the time from the initiation of osimertinib administration to disease progression or death, TTF was defined as the time from the initiation of osimertinib administration to the date of last administration, and OS was defined as the time from the initiation of osimertinib administration to death. The data cutoff date was January 31, 2023. Disease progression was assessed according to the Response Evaluation Criteria in Solid Tumors version 1.1. The incidence and severity of all AEs were documented according to the Common Terminology Criteria for Adverse Events version 5.0.

### Statistical analyses

Survival curves were plotted using the Kaplan–Meier method. Differences in PFS, TTF, and OS between the standard dose and dose reduction groups were assessed using the log-rank test. Univariable and multivariable regression analyses were performed using the Cox proportional hazards model, considering independent factors affecting PFS prolongation. Only factors that showed *p* < 0.05 in the univariable analysis were entered into the multivariable analysis as explanatory variables. *P* values < 0.05 were considered statistically significant. Statistical analysis was performed using IBM SPSS Statistics version 28.0 (IBM, Armonk, NY, USA).

## Results

### Patient characteristics

Of the 88 patients with NSCLC showing advanced *EGFR* mutation or recurring after surgery whose therapy was started with osimertinib as the first-line therapy, three patients with an unknown number of TKI therapeutic days were excluded. Finally, 85 patients, including those with exon 19 deletion (*n* = 35), L858R (*n* = 43), and minor mutations (*n* = 7: exon 20 S768I (*n* = 2), exon 21 L861Q (*n* = 3), exon 18 G719X (*n* = 1), and both exon 21 L861Q and exon 18 G719X (*n* = 1)), were included in the analysis. Histologically, all patients had adenocarcinoma. Table 1 shows the patient characteristics. No significant differences in patient characteristics were observed between the standard dose and dose reduction groups. Patients with exon 19 deletion had a slightly higher proportion of programmed cell death-ligand 1 tumor proportion score (PD-L1 TPS) ≥ 50% (20.0% (7/35)) than those with L858R (11.6% (5/43)) although no significant difference was observed.

**Table 1:** Patient characteristics

		Standard dose (n = 60)	Dose reduction (n = 25)	*p* value
Age (years)	Median (range)	74 (49–89)	76 (46–94)	0.36^c^
Age	≥ 75 years, n (%)	29 (48)	14 (56)	0.64^a^
Sex	Female﻿, n (%)	37 (62)	20 (80)	0.13^a^
BSA	< 1.53 m^2^, n (%)	28 (47)	13 (52)	0.81^a^
Histology	Adenocarcinoma﻿, n (%)	60 (100)	25 (100)	-
Smoking history	Never smoke﻿, n (%)	37 (62)	18 (72)	0.46^a^
	Current or ex-smoker﻿, n (%)	23 (38)	7 (28)	
Stage	Advanced﻿, n (%)	48 (80)	20 (80)	1^a^
	Postoperative recurrence﻿, n (%)	12 (20)	5 (20)	
*EGFR* mutation	Exon 19 deletion﻿, n (%)	25 (42)	10 (40)	0.68^a^
	Exon 21 L858R﻿, n (%)	31 (52)	12 (48)	
	Minor mutation﻿, n (%)	4 (6)	3 (12)	
PD-L1 TPS	≥ 50%﻿, n (%)	10 (22)	4 (27)	0.93^b^
	1–49%﻿, n (%)	17 (39)	5 (33)	
	< 1%﻿, n (%)	17 (39)	6 (40)	
	Unknown﻿, n	16	10	-
Brain metastasis	Positive﻿, n (%)	16 (27)	12 (48)	0.08^b^
	Negative﻿, n (%)	43 (73)	13 (52)	
	Unknown﻿, n	1	0	-
Pleural metastasis	Positive﻿, n (%)	43 (72)	21 (84)	0.28^a^
Liver metastasis	Positive﻿, n (%)	4 (7)	6 (24)	0.06^a^
Bone metastasis	Positive﻿, n (%)	26 (43)	9 (36)	0.63^a^
Albumin (g/dL)	Median (range)	3.7 (2.2–4.6)	3.7 (2.7–4.4)	0.43^c^
NLR	Median (range)	3.6 (1.4–22.2)	3.7 (1.0–14.6)	0.16^d^
ALT (U/L)	Median (range)	14.5 (5.0–62.0)	14.0 (5.0–33.0)	0.52^c^
AST (U/L)	Median (range)	19.0 (8.0–42.0)	20.0 (11.0–39.0)	0.72^c^
Total bilirubin (mg/dL)	Median (range)	0.5 (0.1–1.4)	0.5 (0.2–0.8)	0.73^c^
Ccr (mL/min)	Median (range)	67.9 (8.5–124.0)	63.0 (28.3–146.6)	0.78^c^

*ALT* alanine aminotransferase, *AST* aspartate aminotransferase, *BSA* body surface area, *Ccr* creatinine clearance, *EGFR* epidermal growth factor receptor, *NLR* neutrophil to lymphocyte ratio, *PD-L1 TPS* programmed cell death-ligand 1 tumor proportion score

All *p* values were calculated with the following tests: ^a^Fisher’s exact test

^b^Fisher’s exact test performed except for unknown cases

^c^Mann–Whitney *U* test

^d^Mann–Whitney *U* test performed except for unknown cases (*n* = 5)

Dose reduction was observed in 25 patients (29.4%) within 6 months after osimertinib initiation, and the median time to dose reduction was 1.2 months. Among them, three started the treatment with a reduced dose (40 mg/day) due to their old age (89, 90, and 91 years), and 22 experienced dose reduction due to rash (*n* = 10), diarrhea (*n* = 3), liver dysfunction (*n* = 3), malaise (*n* = 1), heart failure (*n* = 1), paronychia (*n* = 1), nausea (*n* = 1), myocarditis (*n* = 1), and anorexia (*n* = 1).

### PFS

**Fig. 1:**
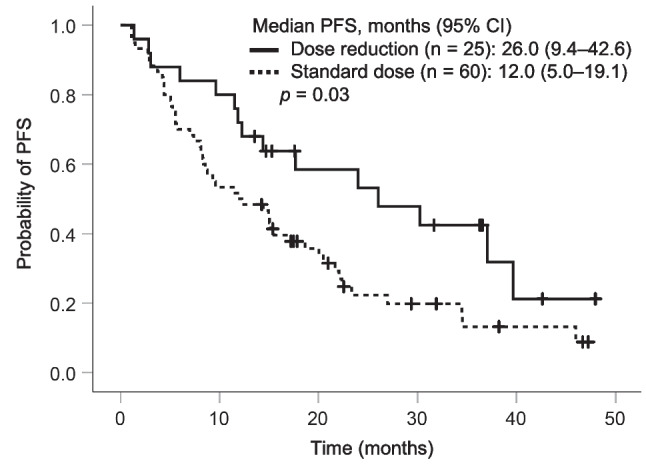
Kaplan–Meier curves of PFS in patients who had dose reduction within the first 6 months and those who remained on osimertinib 80 mg/day. PFS progression-free survival, CI confidence interval

The overall median PFS was 15.1 months (95% confidence interval (CI), 10.0–20.1 months), and the response rates were 57.6% at 12 months and 43.8% at 18 months. The dose reduction group showed significantly greater median PFS than the standard dose group (26.0 vs. 12.0 months, *p* = 0.03) (Fig. 1). The progression rates due to brain metastasis were 4.0% (1/25) and 5.0% (3/60) in the dose reduction and the standard dose groups, respectively. Additionally, the dose reduction group showed significantly greater median PFS than the standard dose group (37.1 vs. 8.4 months, *p* = 0.01) in elderly patients defined as ≥ 75 years old (Supplemental Fig. 1). On the other hand, there was no significant difference in PFS between the dose reduction group and the standard dose group (24.0 vs. 15.2 months, *p* = 0.64) in non-elderly patients defined as < 75 years old.

### Prognostic factors for PFS

A Cox regression analysis was performed to investigate the prognostic factors for PFS in 84 patients, excluding one patient with unknown brain metastasis. The univariable analysis revealed that minor mutation, pleural metastasis, and liver metastasis were significant factors associated with worse PFS, and dose reduction in the first 6 months was a significant factor associated with better PFS (Table 2). The multivariable analysis revealed that pleural and liver metastasis were independent factors associated with worse PFS, and mutation type (exon 21 L858R) and dose reduction in the first 6 months were significant factors associated with better PFS (Table 2).

**Table 2:** Univariable and multivariable analyses of prognostic factors for progression-free survival

Variables	Category	Univariable analysis		Multivariable analysis	
		HR (95% CI)	*p* value	HR (95% CI)	*p* value
Age	≥ 75 years	0.57 (0.53–1.43)	0.87		
Sex	Female	0.81 (0.48–1.37)	0.43		
BSA	≥ 1.53 m^2^	1.06 (0.65–1.76)	0.81		
Smoking history	Never smoke	0.92 (0.55–1.55)	0.75		
Stage	Advanced	1.45 (0.75–2.80)	0.27		
Mutation type	Exon 19 deletion	Ref		Ref	
	Exon 21 L858R	0.66 (0.39–1.12)	0.13	0.47 (0.27–0.84)	0.01
	Minor mutation	4.28 (1.72–10.68)	0.002	2.28 (0.88–5.92)	0.09
Brain metastasis	Positive	1.53 (0.92–2.55)	0.11		
Pleural metastasis	Positive	2.11 (1.08–4.10)	0.03	2.16 (1.10–4.26)	0.03
Liver metastasis	Positive	2.50 (1.22–5.14)	0.01	3.56 (1.60–7.91)	0.002
Bone metastasis	Positive	1.47 (0.89–2.43)	0.13		
Hypoalbuminemia	Grade 0	Ref			
	Grade 1–2	1.73 (0.98–3.05)	0.06		
Dose reduction in the first 6 months	Yes	0.53 (0.29–0.95)	0.03	0.36 (0.19–0.67)	0.001

A multivariable analysis was performed on 84 patients, excluding one patient with unknown brain metastasis

*HR* hazard ratio, *CI* confidence interval, *BSA* body surface area

### TTF and OS

**Fig. 2:**
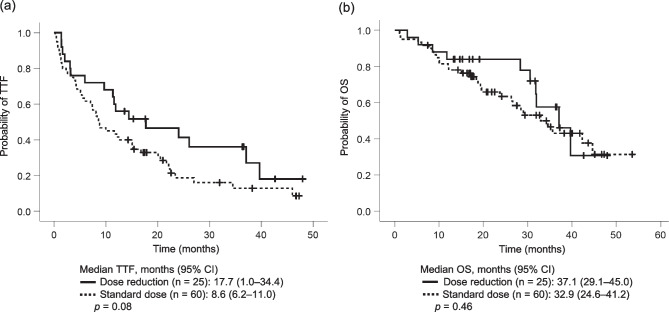
Kaplan–Meier curves of TTF (**a**) and OS (**b**) in patients who had dose reduction within the first 6 months and those who remained on osimertinib 80 mg/day. TTF time to treatment failure, OS overall survival, CI confidence interval

The overall median TTF was 11.5 months (95% CI: 7.8–15.2 months). The median TTF was not significantly different between the dose reduction and the standard dose groups (17.7 vs. 8.6 months, *p* = 0.08) (Fig. 2a). The overall median OS was 34.5 months (95% CI, 28.8–40.3 months), and 82.2% and 69.2% of patients were alive at 12 and 24 months, respectively. No significant difference in the median OS was observed between the dose reduction and the standard dose groups (37.1 vs. 32.9 months; *p* = 0.46) (Fig. 2b).

### Changes in the grade of AEs with dose reduction of osimertinib

Of the 25 patients who had dose reduction within the first 6 months, 18 patients experienced 19 AEs that triggered the initial dose reduction after starting on osimertinib 80 mg/day, excluding 3 patients starting from 40 mg/day due to old age and 4 patients due to the lack of severity data before and after dose reduction. The severity of all AEs improved within 60 days after dose reduction compared with the severity before dose reduction. Grades 3 and 1–2 of AEs were 52.6% (10/19) and 47.4% (9/19) before the dose reduction, and 0% and 15.8% (3/19) after the dose reduction, respectively (Supplemental Table 1).

### Relationship between incidence of ILD and PFS

**Fig. 3:**
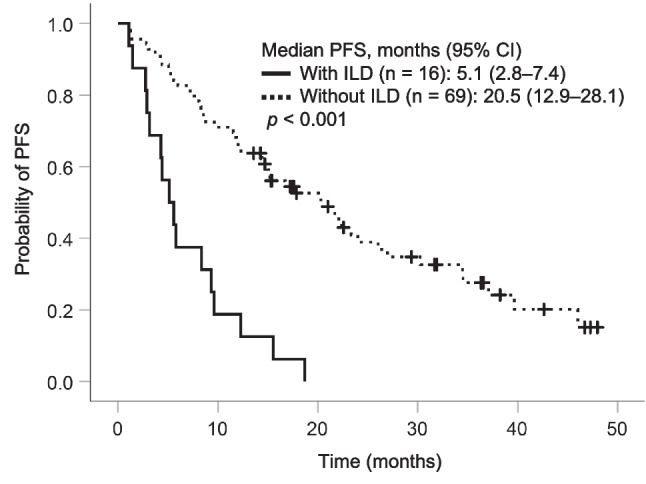
Kaplan–Meier curves of PFS in patients with or without osimertinib discontinuation due to ILD *PFS* progression-free survival, *CI* confidence interval, and *ILD* interstitial lung disease

Eighteen patients in the standard dose group and 6 patients in the dose reduction group developed ILD, and no significant difference in the incidence rate of ILD was observed between the standard dose group and the dose reduction group (30.0% (18/60) vs. 24.0% (6/25), *p* = 0.79). The median time to onset of ILD was 1.6 months in the standard dose group and 5.8 months in the dose reduction group. Due to the onset of ILD, patients in the standard dose group resulted in a higher proportion of the treatment discontinuation compared to those in the dose reduction group (25.0% (15/60) vs. 4.0% (1/25)). Patients in the standard dose group had a lower proportion of the resumption of osimertinib treatment after withdrawal or the continuation without discontinuation after the incidence of ILD compared to those in the dose reduction group (5.0% (3/60) vs. 20.0% (5/25)). The median PFS was significantly shorter in patients with discontinuation due to ILD (*n* = 16) than in those without discontinuation due to ILD (5.1 vs. 20.5 months, *p* < 0.001) (Fig. 3).

On one hand, 32.6% (14/43) of the elderly patients and 23.8% (10/42) of the non-elderly patients developed ILD. In the elderly patients, the treatment discontinuation rate due to ILD tended to be higher in the standard dose group than in the dose reduction group (34.5% (10/29) vs. 7.1% (1/14)). In the non-elderly patients, the treatment discontinuation rate due to ILD in the standard dose group was 16.1% (5/31), although no patient in the dose reduction group discontinued treatment due to ILD.

## Discussion

Osimertinib is widely used in the treatment of *EGFR*-mutated NSCLC. To the best of our knowledge, this is the first retrospective real-world data study evaluating the effect of dose reduction of osimertinib on efficacy in patients of all ages who were started on osimertinib as the first-line therapy for *EGFR*-mutated NSCLC.

The median PFS was significantly prolonged in the dose reduction group compared with that in the standard dose group. This may be due to differences in osimertinib exposure and treatment discontinuation due to ILD between the two groups. A previous study reported that PFS was prolonged in patients with predicted osimertinib trough concentration (C_min, pred_) < 166 µg/L compared with those with C_min, pred_ ≥ 166 µg/L [11]. Additionally, the AURA study that evaluated the safety and efficacy of osimertinib at doses of 20–240 mg once daily showed similar response rates at doses of 20, 40, and 80 mg/day [12]. Furthermore, an observational study of Japanese patients aged 75 years and older treated with osimertinib as the first-line therapy showed that PFS tended to be longer in patients with reduced osimertinib dose than in those with a standard dose (80 mg/day) [13]. These findings are consistent with those of our study. The relative increase in osimertinib exposure due to the administration of the standard dose compared with the reduced dose of osimertinib may contribute to the increased treatment discontinuation rate due to ILD. In this study, although no significant difference in the incidence rate of ILD was observed between the standard dose group and the dose reduction group (30.0% vs. 24.0%), the rate of treatment discontinuation due to ILD was higher in the standard dose group than in the dose reduction group (25.0% vs. 4.0%). Moreover, the patients who discontinued treatment due to ILD showed significantly shorter PFS than the other patients (Fig. 3). Among 16 patients who discontinued treatment due to ILD, 15 patients were in the standard dose group, which may have contributed to the poor PFS in the standard dose group. With first-generation TKI gefitinib, patients who developed ILD tended to show higher exposure to gefitinib than those without ILD [14]. Additionally, it has been reported that pneumonitis tends to increase with increased exposure to osimertinib [15], and increased osimertinib exposure in the standard dose group may have resulted in treatment discontinuation. Therefore, the dose reduction group tended to have a longer median TTF than the standard dose group. In this study, plasma concentrations of osimertinib were not measured, and the association between exposure to osimertinib and ILD onset remains unclear. Furthermore, no significant difference in OS was observed between the groups. Since OS is greatly affected by post-treatment after osimertinib therapy, osimertinib dose reduction in the first-line therapy may not affect OS.

The proportion of patients, who continued or resumed osimertinib treatment after the incidence of ILD because the image findings of pneumonia had resolved and there were no abnormalities in respiratory function, was higher in the dose reduction group (20.0%) than in the standard dose group (5.0%). Treatment discontinuation due to ILD was one patient among six patients with incidence of ILD in the dose reduction group, indicating that ILD observed in patients with dose reduction may not preclude continuation of treatment. On one hand, dose reduction within 6 months significantly prolonged PFS in the elderly patients, whereas it did not significantly contribute to PFS prolongation in the non-elderly patients (Supplemental Fig. 1). This may reflect that the treatment discontinuation rate due to ILD was higher in elderly patients compared to non-elderly patients in the standard dose group (34.5% vs. 16.1%). Since elderly patients have relatively low body surface area, the fixed dose of osimertinib might result in relatively higher blood concentrations in the standard dose group, causing increased severe ILDs and higher rates of osimertinib treatment discontinuation, while there was no relationship between dose reduction and PFS in non-elderly patients. These results suggest that dose reduction of osimertinib with a lower frequency of adverse events may be an option in a wide range of ages, especially in the elderly patients.

Generally, osimertinib can greatly penetrate the blood–brain barrier and has a reduced risk for central nervous system progression compared with first-generation EGFR-TKIs, such as gefitinib and erlotinib [16, 17]. However, dose reduction caused a lower osimertinib concentration in the cerebrospinal fluid, raising concerns about an increased risk of brain metastasis recurrence. In this study, metastasis recurrence was not significantly different between the dose reduction group (4.0%) and the standard dose group (5.0%). In this study, periodic imaging tests to confirm brain metastasis recurrence were not performed in some cases. Therefore, further investigations are needed to evaluate the effect of dose reduction on the exacerbation of brain metastasis.

In the dose reduction group, the severity of AE that triggered the initial dose reduction was grade 3 in more than half of the cases at the time of each AE onset. However, the severity grades of all AEs were reduced to grade 1 or less within 60 days after dose reduction (Supplemental Table 1). Therefore, almost all AEs were appropriately managed by dose reduction.

In this study, the median PFS was 15.1 months, which was shorter than that reported in a multicenter, retrospective observational study on Japanese patients (OSI-FACT study) in which osimertinib as a first-line therapy showed efficacy with a median PFS of 20.5 months [10]. Additionally, the median OS was 34.5 months, which was a little shorter than 38.6 months in the FLAURA study [7]. The reason why the PFS and OS observed in this study were shortened compared with those in previous studies might be related to the high incidence rate of ILD. In this study, the incidence rate of ILD was 28.2% (24/85), which was more than two times as frequent as that in the FLAURA study (12.3%) [5]. Additionally, the discontinuation rate due to ILD was 18.8% (16/85), which was more than 1.8 times as frequent as that in the OSI-FACT study (10.4%) [10]. Previous reports have shown that PFS is significantly shorter, and OS tends to be shorter in patients with ILD than in those without ILD in Japanese patients [18]. The high treatment discontinuation rate due to ILD may have affected the lower efficacy of this study compared with previous reports. In this study, the multivariable analysis revealed that the independent factors for PFS shortening were pleural and liver metastasis. This finding is consistent with a previous report [10]. Additionally, the independent factors for PFS prolongation were exon 21 L858R and dose reduction within 6 months. Generally, exon 19 deletion is associated with greater antitumor efficacy of EGFR-TKIs than exon 21 L858R [19]. PD-L1 TPS ≥ 50% is a poor prognostic factor for osimertinib therapy [10]. In this study, the rate of PD-L1 TPS ≥ 50% in patients with exon 19 deletion was about twice as high as that in patients with exon 21 L858R, which caused reduced efficacy in patients with exon 19 deletion.

This study has some limitations. First, this was a single-center retrospective study with a small sample size. Thus, the results cannot be considered definitive. Second, the performance status of each patient was not available in the electronic medical records. Third, in the multivariable analysis, PD-L1 TPS, which has been reported as a prognostic factor [10], could not be included as an explanatory variable because many cases were unknown.

## Conclusion

Early dose reduction of osimertinib is an effective therapeutic strategy that not only reduces AE severity but also prolongs PFS in patients with *EGFR*-mutated NSCLC.

### Supplementary Information

Below is the link to the electronic supplementary material.

Supplementary file1 (DOCX 65 KB)

Supplementary file2 (DOCX 19 KB)

## Data Availability

No datasets were generated or analysed during the current study.
