# Supplementary material for: Effect of early dose reduction of osimertinib on efficacy in the first-line treatment for EGFR-mutated non-small cell lung cancer
Source: Invest New Drugs. 2024 Mar 27;42(3):281–8. doi: 10.1007/s10637-024-01432-4 (PMC11164814; doi:10.1007/s10637-024-01432-4)
Supplement: Supplementary file 1 — Supplementary file1 (DOCX 65 KB) [file 10637_2024_1432_MOESM1_ESM.docx]

**Supplemental Figure 1** Kaplan–Meier curves of PFS in patients who had dose reduction within the first 6 months and those who remained on osimertinib 80 mg/day in elderly patients (≥75 years old) (**a**) and non-elderly patients (<75 years old) (**b**)

*PFS* progression-free survival, *CI* confidence interval

Article title：

Effect of early dose reduction of osimertinib on efficacy in the first-line treatment for EGFR-mutated nonsmall cell lung cancer

Journal name：

Investigational New Drugs

Author names：

Tomoki Hori, Kazuhiro Yamamoto, Takefumi Ito, Shigeki Ikushima, Tomohiro Omura, Ikuko Yano

Affiliation and e-mail address of the corresponding author：

Kazuhiro Yamamoto, PhD

Department of Pharmacy, Kobe University Hospital, 7-5-2 Kusunoki-cho, Chuo-ku, Kobe 650-0017, Japan

E-mail address: [yamakz@med.kobe-u.ac.jp](mailto:yamakz@med.kobe-u.ac.jp)
