# Supplementary material for: Effect of early dose reduction of osimertinib on efficacy in the first-line treatment for EGFR-mutated non-small cell lung cancer
Source: Invest New Drugs. 2024 Mar 27;42(3):281–8. doi: 10.1007/s10637-024-01432-4 (PMC11164814; doi:10.1007/s10637-024-01432-4)
Supplement: Supplementary file 2 — Supplementary file2 (DOCX 19 KB) [file 10637_2024_1432_MOESM2_ESM.docx]

**Supplemental Table 1** Osimertinib adverse event before and after the dose reduction that triggered the initial dose reduction in patients who had dose reduction within the first 6 months

| Patient no. | Reasons for dose reduction | Before dose reduction | After dose reduction |
| --- | --- | --- | --- |
| 1 | Diarrhea | 3 | 0 |
| 2 | Liver dysfunction | 3 | 0 |
| 3 | Rash | 3 | 0 |
| 4 | Liver dysfunction | 3 | 1 |
| 5 | Rash | 3 | 0 |
| 6 | Rash | 3 | 0 |
| 7 | Rash | 3 | 0 |
| 8 | Liver dysfunction | 2 | 1 |
| 9 | Heart failure | 2 | 1 |
| 10 | Rash | 2 | 0 |
| 10 | Paronychia | 1 | 0 |
| 11 | Rash | 1-2 | 0 |
| 12 | Rash | 1-2 | 0 |
| 13 | Rash | 3 | 0 |
| 14 | Paronychia | 2 | 0 |
| 15 | Rash | 1-2 | 0 |
| 16 | Nausea | 3 | 0 |
| 17 | Myocarditis | 3 | 0 |
| 18 | Rash | 1-2 | 0 |

Each grade after dose reduction indicates the highest grade within 60 days after dose reduction

Cases starting from 40 mg due to old age (*n* = 3) and cases in which grade could not be evaluated before and after dose reduction (*n* = 4) were excluded

Article title：

Effect of early dose reduction of osimertinib on efficacy in the first-line treatment for EGFR-mutated nonsmall cell lung cancer

Journal name：

Investigational New Drugs

Author names：

Tomoki Hori, Kazuhiro Yamamoto, Takefumi Ito, Shigeki Ikushima, Tomohiro Omura, Ikuko Yano

Affiliation and e-mail address of the corresponding author：

Kazuhiro Yamamoto, PhD

Department of Pharmacy, Kobe University Hospital, 7-5-2 Kusunoki-cho, Chuo-ku, Kobe 650-0017, Japan

E-mail address: [yamakz@med.kobe-u.ac.jp](mailto:yamakz@med.kobe-u.ac.jp)
